# The Role of Brain-Derived Neurotrophic Factor in Immune-Related Diseases: A Narrative Review

**DOI:** 10.3390/jcm11206023

**Published:** 2022-10-12

**Authors:** Marcin Sochal, Marta Ditmer, Agata Gabryelska, Piotr Białasiewicz

**Affiliations:** Department of Sleep Medicine and Metabolic Disorders, Medical University of Lodz, 92-215 Lodz, Poland

**Keywords:** BDNF, sleep medicine, immunity

## Abstract

Brain-derived neurotrophic factor (BDNF) is a neurotrophin regulating synaptic plasticity, neuronal excitability, and nociception. It seems to be one of the key molecules in interactions between the central nervous system and immune-related diseases, i.e., diseases with an inflammatory background of unknown etiology, such as inflammatory bowel diseases or rheumatoid arthritis. Studies show that BDNF levels might change in the tissues and serum of patients during the course of these conditions, e.g., affecting cell survival and modulating pain severity and signaling pathways involving different neurotransmitters. Immune-related conditions often feature psychiatric comorbidities, such as sleep disorders (e.g., insomnia) and symptoms of depression/anxiety; BDNF may be related as well to them as it seems to exert an influence on sleep structure; studies also show that patients with psychiatric disorders have decreased BDNF levels, which increase after treatment. BDNF also has a vital role in nociception, particularly in chronic pain, hyperalgesia, and allodynia, participating in the formation of central hypersensitization. In this review, we summarize the current knowledge on BDNF’s function in immune-related diseases, sleep, and pain. We also discuss how BDNF is affected by treatment and what consequences these changes might have beyond the nervous system.

## 1. Introduction

The immune-related disease is a term encompassing conditions caused by impaired immune system functioning, which most commonly results in autoaggression. They include, among others, multiple sclerosis (MS), rheumatoid arthritis (RA), systemic lupus erythematosus (SLE), inflammatory bowel disease (IBD), Hashimoto disease, psoriasis, and systemic sclerosis. Their incidence seems to be on the rise, particularly in highly developed countries [1]. The exact etiology of immune-related diseases remains unclear. Some authors propose, as plausible mechanisms, T-helper cell (Th)1/Th2 or Th17/T-regulatory cell (Treg) imbalance [2,3]. Psychological as well as environmental factors are thought to be also implicated: increased prevalence of these diseases in industrialized societies suggests the importance of lifestyle, stress, and low physical activity. Immune-related conditions also seem to have a neuropsychiatric component.

Interaction between the central nervous system (CNS) and the immune system has gained recognition as a factor in the pathophysiology of immune-related diseases over the last few decades. In general, neuropsychiatric conditions tend to be more prevalent in patients with disorders such as SLE, MS, or autoimmune thyroid diseases compared to the general population [4]. Immune-related conditions might even increase the risk of psychotic disorders [4]. According to a metanalysis conducted by Cullen et al., individuals with pemphigoid, celiac disease, Grave’s disease, and psoriasis have an increased risk of psychotic disorders; however, interestingly, Crohn’s disease (CD), SLE, type 1 diabetes, and ulcerative colitis (UC) do not seem to influence the risk [5].

Authors have suggested that such inconsistencies might stem from differences in the pathophysiology of the mentioned diseases. For example, autoantibody specificity and the diversity of molecular pathways activated during inflammation might have a significant influence on clinical manifestations and the disease’s propensity to affect the CNS. A common genetic component might explain some of the associations seen between neuropsychiatric and immune-related conditions as well. Treatments such as glucocorticoids, commonly used in the treatment of immune-related conditions, have been associated with schizophrenia spectrum disorders; however, they do not explain the aforementioned negative associations.

Currently, the best known and most thoroughly researched are interactions between neuropsychiatric diseases and bowel diseases with the immunological background. A channel of information exchange between the gut microbiota and the brain is the gut–brain axis [6]. The microbiome, together with intestinal mucosal cells, forms a complex system excreting hormones and neurohormones, which affects the organism on both a local and systemic level, including through the excitation of neural endings, by neurotransmitters such as GABA, acetylcholine, or serotonin [7,8,9]. Its dysfunction might contribute to diseases of the digestive tract and psychiatric disorders (for example, schizophrenia or mood disorders commonly observed in this group, such as anxiety and depression) [10,11]. Lifestyle also affects the functioning of the gut–brain axis: psychological stress prompts the release of the corticotropin-releasing factor (CRF). CRF, apart from stimulation of adrenocorticotropic hormone (ACTH) secretion, propels the production of neuromodulators (e.g., serotonin by enterocytes) and cytokines [10]. Interaction between the sympathetic and the parasympathetic activity is also crucial in the modulation of interactions occurring within the gut–brain axis, as it attenuates inflammation through the cholinergic anti-inflammatory pathway [12]. Impairments in this system might result in diseases: as was shown in studies, individuals with IBD tend to have lower parasympathetic activity [13]. Moreover, these patients are five times more likely to suffer from psychiatric disorders than the general population [14]. As one study shows, anxiety/depression is also associated with a more severe IBD course [15].

Neurotrophins might be another possible mediator between immune-related diseases and the central nervous system [16,17]. They are substances responsible for the development, growth, and overall functioning of neurons; however, they might also modulate neurotransmitter release at presynaptic terminals [18,19]. One of them is brain-derived neurotrophic factor (BDNF), a nerve growth factor commonly expressed in the central nervous system; the intensity of expression varies depending on the region as well as concomitant pathologies, e.g., psychiatric disorders or aging [20,21]. BDNF fulfils numerous functions, contributing to neuro- and gliogenesis and regulating synaptic plasticity, as well as inhibitory and excitatory transmission [21,22]. BDNF is vital to learning and memory, and it might also modulate the perception of stimuli, as is the case in chronic pain conditions [21,22,23].

It is estimated that 75% of the circulating BDNF is produced in the brain [24]. BDNF can cross the blood–brain barrier, and retrograde transport from peripheral to the central nervous system probably also occurs [24]. Thus, BDNF concentrations in the central nervous system and blood are related.

Studies have shown that decreased BDNF accompanies conditions such as depression, bipolar disorder, and schizophrenia. Stress might also disrupt BDNF signaling, whereas medications (antipsychotics, antidepressants) can reduce these untoward changes [25]. Sleep disturbances, a ubiquitous comorbidity in both mental and immune-related diseases, also affect the BDNF level: acute sleep deprivation increases whereas insomnia decreases the level of BDNF [26]. Physiological mechanisms behind the effects of sleep (or lack thereof) on BDNF are still unexplained; this subject remains an active area of research.

This paper aims to summarize the role of BDNF in immune-related diseases and factors potentially contributing to their pathophysiology, such as sleep disorders, circadian rhythms, inflammation, and gut–brain signaling. The influence of treatment on BDNF and its role in pain perception will also be discussed.

## 2. BDNF Properties

BDNF was first identified in 1982. It belongs to a family of neurotrophins, sharing ca. 50% amino acids (aa) with other members of the group: nerve growth factor (NGF), neurotrophin (NT) 3, and NT-4/5 [27]. The gene encoding BDNF is located on chromosome 11p [27]. It contains five exons, with one 3′ exon coding for the mature BDNF protein [27]. The remaining four 5′ exons have tissue-dependent expression, with exons I-III predominantly transcribed in the brain (mainly the cortex and the hippocampus, but also the neocortex, microglia, thalamus, hypothalamus, piriform cortex, amygdala, claustrum, and the dorsal horn of the spinal cord) and exon IV in the lungs and the heart [27,28].

BDNF is synthesized as 247-amino-acid (aa) preproBDNF (32–35 kDa) in the endoplasmic reticulum [29]. This BDNF isoform can be divided into three parts: positions 1–18 (18 aa) constitute the signal sequence (the pre- region), positions 19–128 (110 aa) the pro-domain, and positions 129–247 (119 aa) the mature, final form [29].

Following translocation to the Golgi apparatus, the first 18 aa are cleaved, and metabolically active proBDNF (28–32-kDa) is formed [29].

Further processing might take place intra- or extracellularly. In the former pathway, 110 aa are cleaved from BDNF in the intracellular vesicles by convertases or furin [29]. In the extracellular pathway, proBDNF is split by plasmin or other enzymes, such as metalloproteinase 2 and 9 [29]. The expression of metalloproteinase 9 in particular might be increased by pro-inflammatory cytokines, especially tumor necrosis factor (TNF) [30,31]. Both pathways produce mature BDNF (~13-kDa), which acts on the tropomyosin kinase B receptor (TrkB) pathway [32,33]. TrkB acts as a tyrosine kinase, dimerizing and phosphorylating one another in response to BDNF. This leads to the activation of pathways such as the MEK/mitogen-activated protein kinase (MAPK) pathway, extracellular signal-regulated kinase, the PI3K/Akt/mammalian target of rapamycin (mTOR) pathway, and the phospholipase C gamma pathway [34]. The PI3K/Akt/mTOR signaling might be of special importance to the neuroprotective BDNF effects. mTOR is thought to stimulate autophagy, among others, via the ribosomal protein S6 kinase (p70S6K) [35]. As Chen et al. have shown, activation of this pathway by BDNF protects neurons from damage caused by hypoxia [35]. Moreover, activation of PI3K/Akt/mTOR via huperzine was found to protect neurons from oxidative glutamate damage [36].

The BDNF/TrkB pathway might stimulate synaptic plasticity through modulation of its functional and structural aspects. BDNF has been shown to increase synaptic density: in a study on mice, BDNF knock-out specimens had decreased preganglionic synaptic innervation density to sympathetic neurons [37]. The BDNF/TrkB pathway might also contribute to the increase in spine density caused by prolonged BDNF exposure [38]. In another study, activation of the BDNF/TrkB pathway enhanced dendrite arborization [39,40,41,42]. BDNF has also been proven to modulate dendrite branching through TrkB and MAPK pathway-mediated regulation of cypin expression [43].

In regard to the functional aspect, BDNF interacts with signaling pathways associated with different neurotransmitters. One of them is glutamatergic transmission. On a presynaptic level, in nerve terminals, BDNF is able to modulate the amount of glutamate released to the synaptic cleft through stimulation of [35] mitogen-activated protein kinase-dependent synapsin I phosphorylation [44]. Similarly, in cortical astrocytes, BDNF has promoted glutamate release, perhaps via an increase in the intracellular Ca2+ concentration caused by TrkB activation [45]. On the other hand, the BDNF/TrkB complex has been shown to interact with two postsynaptic glutamate receptors: N-methyl-d-aspartate (NMDAR) and α-amino-3-hydroxy-5-methyl-4-isoxazolepropionic acid receptor (AMPAR). It has prompted the phosphorylation of the two NMDAR subunits, as well as increased two AMPAR subunits [46,47,48].

TrkB also stimulates angiogenesis and endothelial survival, which is important in recovery from injuries. The TrkB pathway has been associated with memory acquisition and learning; its dysfunction has been linked to diseases such as Alzheimer’s disease (AD) [49]. It is worth mentioning that BDNF might not be the sole contributor to the effects of TrkB activation, as other neurotrophins, such as NT-4/5, might also act on the TrkB pathway [50].

p75NTR is a transmembrane glycosylated receptor [51]. Since, in itself, it has no enzymatic activity, in order to exert a biological effect, it recruits downstream proteins such as Trks, Nogo, and myelin-associated glycoprotein (MAG) [34].

proBDNF, via the p75NTR pathway, might exert opposite effects to BDNF, promoting synaptic elimination and decreasing the likelihood of firing an action potential in the postsynaptic neuron via decreasing the amplitude of a field excitatory postsynaptic potential (an indicator of synaptic strength) [52]. Through the c-Jun N-terminal kinase (JNK) pathway associated with this receptor, it might trigger apoptosis [28]. Inhibition of the p75NTR pathway has a neuroprotective effect [52]. 

**Figure 1:**
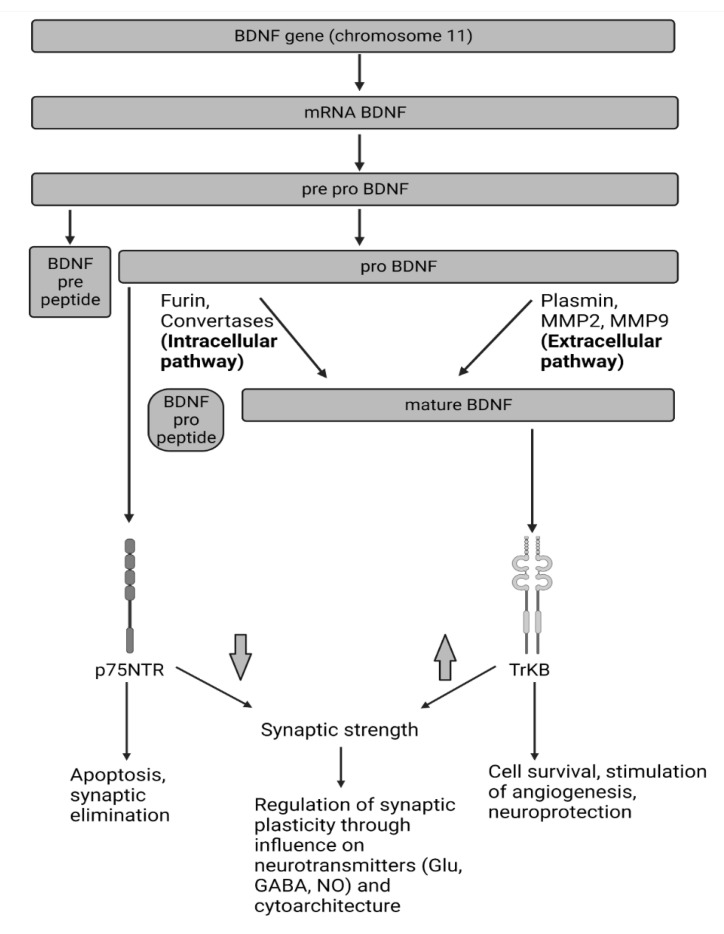
BDNF signaling pathways. BDNF is translated as its isoform, preproBDNF. In the Golgi apparatus, preproBDNF is cleaved, which produces proBDNF. ProBDNF might be split into pro-BDNF peptide and mature BDNF intracellularly (with furin or convertases) or extracellularly (with plasmin, MMP2, or MMP9). ProBDNF is metabolically active; it binds to the p75NTR receptor with high affinity, which might cause apoptosis or promote synaptic elimination. Activation of TrkB might result in the stimulation of angiogenesis and neuroprotection, as well as promoting cell survival. Both receptors modulate the synaptic strength, which allows for the regulation of synaptic plasticity. BDNF: brain-derived neurotrophic factor; GABA: gamma-aminobutyric acid; Glu: glutamate; mRNA: messenger RNA; MMP2/9: metalloproteinase 2/9; NO: nitric oxide; p75NTR: p75 neurotrophin receptor-mediated signaling pathway; TrKb: tropomyosin-related kinase.

Figure 1 summarizes discussed BDNF signaling pathways.

## 3. BDNF in Immune-Related Diseases

BDNF has been studied in numerous conditions with different pathological backgrounds. In the rat model of acute pancreatitis (AP), it has been shown that the mRNA level of BDNF was increased after one week following the induction of AP with L-arginine [53]. The role of BDNF is very well understood in the context of colorectal cancer, where it is considered a potential therapeutic target. Tanaka et al. have shown that higher mRNA expression of BDNF and its receptor TrkB is associated with a worse prognosis in colon cancer [54]. Lembo et al. demonstrated that the serum level of BDNF may be helpful in differentiating patients with irritable bowel syndrome and healthy controls (HC) [55]. However, its role in many immune-related conditions is not fully clear.

### 3.1. BDNF in IBD

IBD is a condition encompassing CD and UC. Both are characterized by chronic digestive tract inflammation; they have a relapsing–remitting course. The relationship between BDNF and diseases such as IBD is multifaceted and poorly understood. Authors suggested that neurotrophic factors have an essential role in intestinal inflammation [56]. BDNF might also be associated with IBD comorbidities: a recent meta-analysis demonstrated that patients with IBD have an increased risk of developing Parkinson’s disease, in which BDNF plays an important role and is extensively studied [57,58].

In the gastrointestinal tract, receptors for both BDNF and pro-BDNF are common in neurons of the myenteric and submucosal plexus, as well as mucosal endocrine cells in humans [59,60,61]. The percentage of cells expressing receptors for a specific neurotrophin varies depending on the location: TrkB was displayed on 26 ± 0.6% of endocrine cells in the duodenum and 78 ± 3% for the sigmoid colon; p75NTR was present only in 21% of the ileal endocrine cells [60].

BDNF fulfills essential functions in the gastrointestinal system. In the UC mouse model, inhibition of the TrkB-PLC/IP3 pathway resulted in a blocked enterocyte cycle and a greater rate of apoptosis in a mice colitis model [62]. Moreover, the study group experienced a more severe course of the disease (as assessed by disease activity, colon mucosa, and tissue damage indexes) and altered cytokine profile due to elevated expression of IL-4,8 and down-regulated IL-10, which created a pro-inflammatory milieu [62]. In one study, CD patients had higher serum BDNF than healthy controls [61]. Moreover, in this group, the BDNF level was correlated with the severity of insomnia [61]. These findings are essential, as sleep problems in IBD are associated with a more aggressive disease course, which puts patients at a higher risk of surgery and hospitalization [63].

**Figure 2:**
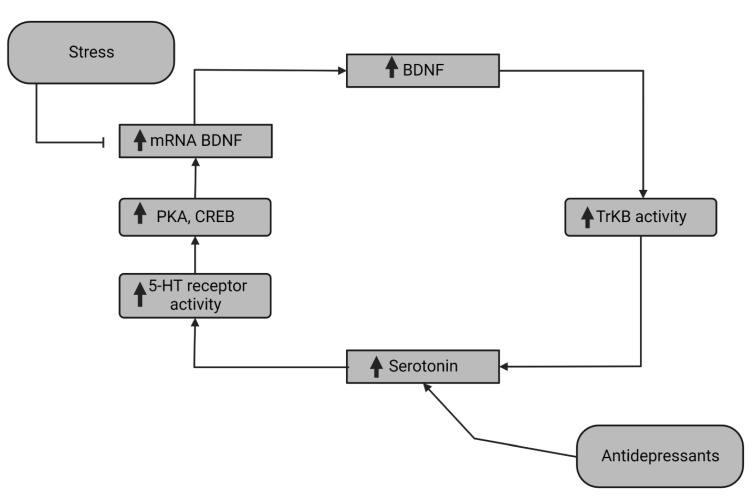
Mutual interactions between BDNF and serotonin. Antidepressants increase the level of serotonin, which accounts for higher 5-HT receptor activity. Activation of this receptor increases the cAMP level, which in turn induces the PKA/CREB signaling pathway and stimulates BDNF expression. Stress, on the other hand, hinders the production of this neurotrophin. 5-HT: serotonin; BDNF: brain-derived neurotrophic factor; CREB: cAMP response element-binding protein; mRNA: messenger RNA; PKA: protein kinase A; TrKb: tropomyosin related kinase.

One of the ways that BDNF might affect the gastrointestinal system is through its interaction with serotonin and serotonin transporters. Figure 2 shows a simplified scheme of this relation in the organism. However, this subject has been widely studied in the CNS, but not the enteric nervous system (ENS).

In the CNS, BDNF might promote the development of the serotonergic neurons through the TrkB pathway. There could also be a feedback loop between BDNF and serotonin. Activation of serotonin receptor 1A (5HT1A) leads to an increase in cAMP, which activates PKA and cAMP response element-binding protein (CREB) [64,65]. This results in an upregulation in BDNF production. It could be hypothesized that such processes take place outside of the CNS. However, this subject requires further studies.

BDNF, on the other hand, has been shown to stimulate serotonin production, thus promoting the peristaltic reflex [59]. Moreover, in mice, BDNF-deficient specimens had a lower number or activity of serotonin receptor 1A (5HT1A), further indicating mutual regulation between both BDNF and serotonin signaling [66].

Serotonin signaling might have a notable role in the development and course of IBD: studies show that inhibition of 5-HT7 might lead to a reduction in inflammation [67]. As another study shows, in Crohn’s ileitis, Crohn’s colitis, and ulcerative colitis, serotonin reuptake is inhibited [68]. Selective serotonin reuptake inhibitors (SSRI), a class of antidepressants that increase the concentration of the mentioned neurotransmitters in synapses, were shown to affect the IBD course positively. Patients administered SSRIs had a significantly lower risk of uncontrollable disease exacerbation, which would require therapy intensification [69].

BDNF might also act on enteric glia, inhibiting apoptosis, as they express both p75NTR and TrkB receptors [70]. The TrkB receptor was proven essential to these cells’ functioning during their development and maintenance. Enteric glial cells fulfill a similar function to those in the nervous system; however, apart from cytoprotective and trophic effects, they also maintain the intestinal barrier, probably through interactions with the epithelium [71,72]. According to the authors, impairment of the enteric glial cells’ function might contribute to the IBD pathophysiology [71].

### 3.2. BDNF in MS and Experimental Autoimmune Encephalomyelitis

Multiple sclerosis is a disease characterized by neuroinflammation, demyelination, gliosis, and axonal loss; experimental autoimmune encephalomyelitis (EAE) approximates the aforementioned main pathological mechanisms of MS. BDNF might play a role in these conditions.

BDNF in the CNS is primarily produced by neurons and, in smaller amounts, by the microglia, astrocytes, oligodendrocytes, and infiltrating immune cells [73,74]. In mice with BDNF deletion restricted to the CNS, BDNF produced by the immune cells alone did not alleviate disease severity [75]. Thus, it seems that BDNF produced in the CNS has a primary role in the pathophysiology of MS and EAE.

Some authors suggest that BDNF might participate in the modulation of neuroinflammation, among others, by interacting with the transcription factor NF-κB [76]. They can mutually induce each other’s expression. However, the exact pathways behind this regulatory influence remain elusive. Marini et al. have shown that the BDNF promoter contains the NF-κB binding site [77]. Moreover, NF-κB was necessary to increase BDNF expression and exert neuroprotective effects after N-methyl-d-aspartate (NMDA) activation [77]. Xu et al. have also found a κB binding site in the BDNF promoter and demonstrated the significance of myeloid differentiation primary response 88 (MyD88)/NF-κB signaling in the induction of BDNF expression [78]. MyD88 is an adaptor protein, which binds Toll-like receptors (TLR) (except for TLR3) stimulated by a ligand. It activates transcription factors, such as the mentioned NF-κB or interferon regulatory factors, which facilitates the further development of inflammation. Thus, BDNF can potentially modify the response to immune challenges.

According to Giaccobo, activation of the TrkB pathway by BDNF leads to the phosphorylation of IκBα (NF-κB inhibitory unit), which allows for proteasomal degradation, producing NF-κB [76]. Migration of this unit to the nucleus results in an increase in the transcription of genes coding for proteins participating in inflammation [76]. Cell survival, proliferation, and apoptosis might be affected by this transcription factor as well. NF-κB is, in general, considered to exert an antiapoptotic influence; however, it might also contribute to apoptosis, as is the case in oxidative injury [79].

BDNF has also been implicated in the process of remyelination. Astrocytes were observed to have increased BDNF production following demyelinating insult in MS and EAE [80]. This neurotrophin is also actively expressed at the site of actively demyelinating lesions in the MS, particularly in the early stage of their development [81]. Studies on animal models of demyelination have shown that decreased BDNF production in heterozygotes results in impaired remyelination processes [80]. BDNF, in this aspect, exerts its effect primarily through TrkB; p75NTR does not seem to be associated with myelination in the CNS [80]. Fletcher et al., in their study, targeted the TrkB of myelin-producing oligodendrocytes with a molecule mimicking the TrkB-binding region of BDNF [82]. Following demyelinating insult, they observed an increase in myelin sheet thickness, oligodendrocyte differentiation, and higher prevalence of myelinated axons, which shows the great potential of targeting pathways associated with BDNF in MS therapy [82].

Studies on the changes in serum BDNF levels in MS are conflicting, showing both higher and lower levels in comparison with healthy controls [24,83]. Some authors suggest that the method of measurement as well as disease duration might account for this variability [84]. Interestingly, recent studies have shown that in patients with relapsing–remitting MS, the BDNF concentration is elevated in the relapse phase [83]. This increase was associated with the restitution of lesions [81,83].

The role of BDNF in the cognitive decline accompanying this disease is not clear. One means of studying this topic is to compare individuals with Val66Met polymorphism, which lowers BDNF production by ca. 18–30%, with the general population.

Some authors observed a higher volume of gray matter (GM) in the brains of Val66Met carriers, while Liguori et al. obtained the opposite results—Val66Met individuals are at a higher risk of GM atrophy, which could be associated with neuropsychological impairments. On the other hand, Mero et al. have concluded that BDNF gene polymorphism does not influence cognitive faculty in the Norwegian population [85]. In a recent study, Portaccio et al. have revealed that this polymorphism might actually preserve cognitive function in MS patients: Val66Met carriers achieved better results on the Rao’s Brief Repeatable Battery and the Stroop Color Word Test [85]. These findings are highly interesting and unexpected, since a lower BDNF level is usually associated with cognitive impairment [86]. Moreover, Met66Val polymorphism itself is perhaps associated with a higher risk of AD development [87]. The authors of the study suggested that this anomalous influence might be ascribed to the pathophysiological mechanism of MS, such as neuroinflammation, a high BDNF concentration, subsequent p75NTR activation, and apoptosis, as well as glutamate excitotoxicity [85].

### 3.3. BDNF in Spondyloarthritis and RA

Both spondyloarthritis (SpA) and RA are common chronic inflammatory diseases of the joints, leading to the progressive degeneration of the affected tissues. SpA is an umbrella term for a number of conditions, such as enteropathic arthropathies, psoriatic arthritis, or ankylosing spondylitis, whereas RA is a systemic disease, affecting mainly peripheral synovial joints.

BDNF might play a significant role in these conditions. Grimsholm et al. have shown that mice induced with arthritis had, in contrast with controls, BDNF and p75 in inflammatory infiltrates of the affected joints, as well as higher p75, BDNF, and TrkB expression in articular chondrocytes [88]. Notably, in more severe arthritis cases, p75 levels were lower [88]. Similarly, in humans, p75NTR expression might be upregulated in the synovial fluid of patients with SpA and RA [89]. In this study, expression of TrkB was also higher in the RA group [89]. Results of BDNF expression levels for both SpA and RA groups as well as TrkB expression in the SpA group did not reach statistical significance, perhaps due to the small sample size (9 RA and 16 SpA patients) or the use of osteoarthritis instead of healthy subjects as a control group [89]. Indeed, in other studies, authors showed that BDNF might be found in the synovial fluid of certain RA patients but never in healthy controls; similarly, BDNF was found to be upregulated in the synovial fluid of SpA patients [88,90]. According to Cheon et al., a negative correlation was found between BDNF levels, depression, and disease severity in RA patients [91]. However, another study showed that, in RA, BDNF levels in synovial tissue do not correlate with inflammatory markers, such as ESR, WBC, number of infiltrating inflammatory cells, or TNF level [92]. In this study group, a decrease in plasma BDNF following anti-TNF treatment also did not correlate with the disease activity score or ESR [92]. These results indicate that BDNF, perhaps with other neurotrophins, might affect inflammatory processes in affected joints; however, its influence on the disease severity remains disputable. Through p75NTR, it might be able to stimulate apoptosis, but in order to exert this effect, its concentration needs to be appropriately high.

Taken together, regulation of regional inflammatory processes/disease severity does not seem to be the primary BDNF role in the discussed conditions. It is possible that BDNF in SpA and RA exerts mainly a neuroprotective effect, as well as participating in the modulation of pain: p75NTR was present in nerve fascicles and the center of sensory corpuscles in non-inflamed joints but was significantly higher in inflamed ones [88,92]. Similarly, BDNF was detected in the nerve fascicles of the inflamed joint but not in healthy ones [88].

Neurotrophins can also exert an effect on blood vessels, affecting the supply of oxygen, nutrients, and, in consequence, disease progress. In RA patients, synovium was negative for p75NTR and TrkB. However, p75NTR was observed in the outer parts of arterioles in biopsy material of OA as well as RA patients [92]. TrkB, on the other hand, was not present in blood vessel walls but in perivascular nerve fibers [92]. In SpA, p75NTR was present in the sublining layer and the endothelium and correlated with lymphoid aggregates [90]. Authors ascribed a relatively higher level of p75NTR in this group compared with the RA group to a higher degree of vascularity in SpA.

It is difficult to determine whether BDNF exerts different effects in MS and SpA. According to studies, in RA serum, the BDNF level might be upregulated in comparison with healthy controls, whereas in SpA, the level of this neurotrophin was significantly decreased [90,92]. However, here, it is important to note that these studies were conducted on different populations; thus, the number of individuals with different genetic predispositions to a baseline level of BDNF production, such as the commonly met polymorphism Val66Met, might affect the study’s outcomes.

### 3.4. Summary

In summary, BDNF might participate in the pathophysiology of immune-mediated diseases in a variety of ways, both directly and indirectly, affecting different signaling pathways. It fulfills an essential role in the modulation of inflammation and exerts a neuroprotective effect. The potential pro-inflammatory properties of BDNF exerted via the NF-κB signaling pathway should further be studied as a therapeutic target. Moreover, extensive research in this area would allow for a better understanding of the connections between pain related to inflammation, BDNF, and sleep disturbances.

## 4. BDNF and Sleep

The circadian rhythm, an internal coordinator of the sleep/wake cycle and numerous other physiological processes, is composed of a set of semi-autonomous clocks [93]. Clocks are regulated through the expression of genes, which form feedback loops, creating an oscillator. This allows for the maintenance of a relatively stable, fixed pattern of function.

Sleep is a natural physiological state necessary for normal functioning and overall survival, governed by the circadian rhythm. It is an active process involving specific brain, metabolic, and endocrine patterns. Sleep consists of non-rapid eye movement (NREM) and rapid eye movement (REM) phases, which cycle throughout the duration of this state. NREM consists of three stages; stage 3 is described as slow-wave sleep. Slow-wave activity (SWA) is a pattern of brain activity characterized by EEG power between 0.5 and 4 Hz, occurring primarily during the aforementioned stages.

BDNF has been evidenced to have a mutual relationship with the circadian rhythm [94]. Both BDNF and TrkB are expressed in the suprachiasmatic nucleus (SCN), the central master clock in mammals, as well as in other brain regions [95]. TrkB might be particularly important for the circadian rhythm, as activation of this receptor was shown to follow a circadian pattern, taking place primarily during subjective night (phase determined as night by the circadian rhythm, characterized by melatonin secretion, low activity level, etc.) [96]. BDNF via TrkB might affect the structural plasticity of the SCN, thus affecting its ability to be influenced by stimuli [95].

Expression of BDNF protein and its mRNA shows dependence on the circadian rhythm: the level of mRNA and BDNF protein is elevated during biological night and day, respectively [95]. However, plasma BDNF, due to its multiple sources, appears to have a relatively tenuous connection to the circadian rhythm [95]. Since BDNF is also stored in platelets, it is possible that studies using serum instead of plasma would yield different, more accurate results regarding diurnal BDNF level variation. Sex might also affect circadian BDNF variability, with women showing more diverse results than men [95].

As one study shows, injecting BDNF into brain ventricles leads to increased REM and NREM duration in rabbits [97]. The same procedure performed on rats yielded increases only in NREM [97]. Interestingly, in a study on heterozygous BDNF knockdown mice (BDNF decreased 50% in comparison with wild-type controls), the experimental group showed a lower number of REM episodes, reduced time spent in REM, and prolonged REM initiation; no changes in NREM were observed [98]. In humans, a reduction in BDNF caused by Val66Met polymorphism has been connected with decreased sleep intensity, time spent in slow-wave sleep, and delta activity, and changes in SWA architecture: slower beginning and longer dissolution [99]. In patients with major depressive disorder, elevated BDNF was related to increased NREM quality and SWA [94].

The mechanism through which BDNF can modulate NREM is unclear, with some authors claiming that it altogether has little impact on SWA [100]. Effects exerted by BDNF on slow-wave sleep appear to differ depending on the location [97]. Intracerebroventricular administration reduces slow-wave activity, whereas intracortical injection leads to a localized increase in this parameter [97]. This could be explained by the different distributions of injected BDNF; in the case of the former mode of administration, it could have impacted other brain structures responsible for the control of sleep (basal forebrain, hypothalamus) [101]. Authors suggest that BDNF controls SWA through its influence on synaptic potentiation during wakefulness [26]. TrkB.T1 (truncated isoform without the tyrosine kinase domain) knock-out mice did not show any changes in NREM sleep, which indicates that the BDNF/TrkB pathway might not be involved in the regulation of this sleep phase [97].

BDNF might exert a strong influence on REM drive through TrkB. TrkB.T1 knock-out mice showed a decrease in REM sleep latency and sleep continuity, and an increase in REM sleep time was observed [97]. However, a similar study involving blockade of TrkB in vivo yielded different results, namely reduced time spent in REM and reduced REM transitions [102]. As another study shows, in heterozygous BDNF knockdown mice after isolated REM sleep deprivation, animals did not have rebound REM, which indicates a lack of homeostatic REM drive [98].

TrkBs are present in regions crucial to the modulation of REM sleep: GABAergic and glutamatergic neurons in the midbrain and brainstem [102]. In this context, the BDNF/TrkB pathway appears to have a particularly important role in the pedunculopontine tegmentum (PPT), which has been demonstrated to be indispensable in the development of the REM drive [98].

Apart from promoting REM, BDNF might be necessary for memory consolidation in this stage. TrkB downstream activates the Ca2+/calmodulin-dependent protein kinase II (CAMKII), which integrates and cements changes in synaptic connections that occur due to long-term potentiation by, among others, inducing changes in genes’ expression [103]. One example of such a gene is Arc/Arg3.1. It is activated by CAMKII and might affect the cytoskeleton of a cell [104]. It is also known that mutation in CAMKII, which impairs protein synthesis in the dendrites, precludes long-term potentiation (LTP) [105]. However, the subject of transcriptional changes occurring in the late stages of LTP remains an active area of research [105].

CAMKII is also necessary in the early stages of LTP; it moves to the synapse and binds to the NMDA glutamate receptor, which in turn potentiates the AMPA glutamate receptor transmission, as well as increasing the number of AMPA receptors in the synapse [105]. These processes are necessary for synapse specificity [105].

BDNF has an interesting relationship with sleep deprivation (SD).

Sleep deprivation, total or partial, appears to increase hippocampal, cortical, and serum BDNF [103]. This effect was not observed after REM SD (also known as paradoxical SD); however, studies’ results vary, with some showing an increased level of BDNF in the brainstem after REM SD [103,106]. Ketamine has similar effects, upregulating BDNF and improving depressive symptoms (decreasing Hamilton Depression Rating Scale score) [26]. Antidepressants and electroconvulsive therapy bring about comparable changes, albeit within weeks, not hours, following administration [26]. Chronic sleep insufficiency, as observed in, e.g., insomnia, has been correlated with decreased BDNF levels [26]. Slow-wave activity appears to be unaffected in insomnia patients. However, cognitive–behavioral therapy and hypnotic medications were shown to increase it [103,107].

It is unclear why chronic sleep restriction decreases whereas sleep deprivation increases BDNF levels. Sleep deprivation and insomnia might be perceived as acute and chronic stress triggers, respectively. Moreover, the latter is strongly related to stress-related mood disorders, such as anxiety disorders or depression; it also renders individuals more prone to stress, which further enhances this effect [108,109]. Acute and chronic stress has been associated with BDNF increase and decrease, respectively; thus, it is possible that BDNF levels change accordingly in insomnia and sleep deprivation [110,111]. An increased level of BDNF might facilitate the formation of new synaptic connections, improving an individual’s coping abilities and problem-solving skills, which plays a greater role in temporary rather than prolonged distress [111].

A positive correlation between BDNF, pain severity scores, and sleep insufficiency assessed by questionnaires suggests that BDNF might act as a mediator between pain and sleep loss [26,112]. Xue et al. have observed that 24-h sleep deprivation before surgery led to an increase in BDNF while lowering the pain threshold [106]. Diseases characterized by chronic sleep insufficiency, such as insomnia, tend to feature low levels of BDNF. However, individuals affected with these conditions report more severe pain as well as a longer pain duration [113].

Obstructive sleep apnea (OSA) is an interesting and interdisciplinary disorder. It is characterized by airway collapse and subsequent airflow obstruction, causing sleep fragmentation: the patient experiences multiple recurring short-term awakenings to take a breath; they tend to remain below the level of consciousness. OSA is associated with chronic intermittent hypoxia, which might exert a far-ranging influence on the organism, affecting, among others, immune functions (OSA is a risk factor for immune-mediated diseases, such as RA or psoriasis) and contributing to neurodegeneration, which develops in the course of this condition [114]. Studies show that hypoxia can induce BDNF expression via the hypoxia-induced-factor-1 pathway [115]. Indeed, OSA patients have a higher level of serum BDNF [116]. In this case, a high BDNF level might exert neuroprotective effects, minimizing the deleterious effects of hypoxia on cells. This is in line with observations made in the mentioned study, as the BDNF level was correlated with cognitive function, as assessed by Montreal Cognitive Assessment (MoCA) questionnaire [116].

Sleep appears to have a close association with immune-related diseases. Multiple epidemiological studies have shown that sleep disorders increase the risk of conditions such as RA, AS, SLE, etc. [114,117,118,119,120]. Moreover, as Ananthakrishnan et al. have revealed, sleep disorders might be associated with a higher risk of relapse in CD, thus modifying the disease course [121]. The discussed neurotrophin could be one of the factors mediating the interactions between sleep disorders and immune functions. As described in detail in this review, BDNF is associated with sleep on multiple levels, from modulating the sleep structure itself to participation in the pathophysiology of mental disorders featuring disrupted sleep, such as depression or anxiety, which accompany immune-related conditions [122]. Conversely, the serum and tissue levels of this neurotrophin can depend on the disease activity and change during its course; BDNF might also directly influence inflammatory processes and control cell apoptosis.

As of now, multiple aspects of interactions between BDNF, sleep, and autoimmunity, e.g., the influence of inflammation or sex on the circadian rhythmicity of BDNF production, remain elusive. However, due to the large body of evidence on its importance to the immune system and circadian rhythm, it is reasonable to study this subject further.

Since sleep disorders, such as OSA, are highly prevalent in modern societies, their association with BDNF needs to be more extensively investigated. They pose a significant burden for the patient due to comorbidities and the progressive loss of cognitive function.

## 5. BDNF as a Pain Mediator

Pain in immune-related diseases can be a response to noxious stimuli occurring at the site of the lesion or persist after the attenuation of inflammation in chronic form. It is one of the symptoms and often the primary complaint of patients suffering from these conditions [123,124,125]. In IBD, it is estimated that more than 70% of patients experience pain, in psoriasis 42.6%, 65–75% in Sjogren syndrome, 40% in Parkinson’s disease, and 49.3% in SpA [125,126,127,128,129]. Nearly every fourth SLE patient might suffer from subjectively strong pain related to the disease manifestations [130]. Chronic pain is a major burden, associated with an overall decline in quality of life, as well as impaired functioning in social and work-related environments [131]. Thus, advances in the field of antinociceptive treatment would be greatly appreciated by this group of patients.

BDNF has been evidenced to be important to nociception, participating in central hypersensitization and the transmission of nociceptive signals in spinal and supraspinal pathways [50]. As one study shows, anti-BDNF antibodies were able to alleviate pain caused by an injury [98].

The sensation of pain requires at least a few elements to take place: first-order neurons in the dorsal root ganglia (DRG), second-order neurons in the spinal dorsal horn, and third-order neurons projecting from the ventrolateral nucleus of the thalamus to the sensory cortex [132]. Interaction between the pre-frontal cortex (PFC) and somatosensory cortices plays a role in the further processing of the signal [132]. PFC connects to various areas of the brain, such as the anterior cingulate cortex, insular cortex, and the thalamus, allowing for the discrimination of pain based on its localization and intensity [132]. The limbic system, another structure connected to the PFC, is responsible for the emotional aspect of pain perception [132].

BDNF can be produced by nociceptors, primarily those that are TrkA-positive [132]. Their projections can terminate in the Lamina I/IIo of the dorsal spinal horn, an important region for the transmission and modulation of pain signals, with various interneurons, inhibitory fibers, and P-substance-containing C fibers [132]. At the spinal level, BDNF participates in relying on signals through the control of glutamate release—an increase in BDNF is associated with an increase in glutamate and glutamatergic transmission via the TrkB/Src/PLC-γ1 pathway [132,133]. Glutamate, a primary excitatory neurotransmitter, is crucial to transmitting nociceptive signals from peripheral receptors.

BDNF does not seem to affect the input to the spinal cord from peripheral receptors. In a study performed by Sikander et al., the evoked potential and the total number of neuronal firings to painful thermal and mechanical stimuli did not differ between wild-type mice and BDNF knock-out specimens [23].

BDNF might facilitate spinal reflexes, perhaps through promoting the NMDA-dependent synaptic facilitation of excitatory postsynaptic current (EPSC) in the neurons of lamina II and blockade of the KCC2 potassium–chloride channel, which decreases GABA signaling [28,134]. However, in the previously mentioned study by Sikander et al., there were no differences in reflex response to acute pain between BDNF knock-out and wild-type specimens, but in a similar study, BDNF knock-out mice had a higher pain threshold in a hotplate test, which involves a reflex from the bulbospinal pathway [23].

In summary, as Sikander et al. have demonstrated, BDNF does not play a major role in processing acute pain, even though it might to some extent modify the reflex response to noxious stimuli.

Another aspect of BDNF’s influence on pain is central sensitization. This process is responsible for both hyperalgesia and allodynia (exacerbated response to painful and innocuous stimuli, respectively) and occurs due to strengthening and increasing the efficacy of synaptic communication between neurons, primarily nociceptive afferents connected to the spinal dorsal horn via small, unmyelinated C fibers. It involves reshaping neuronal circuits, increased excitability of neurons, decreased synaptic inhibition, and alterations in gene expression and protein phosphorylation [135,136]. Due to the aforementioned processes, painful stimuli might come from sources such as low-threshold cutaneous mechanoreceptors with myelinated Aβ fibers [136].

It is primarily involved in chronic pain, persisting after the elimination of a noxious stimulus; however, certain characteristics of central sensitization are present in individuals during the acute stage of inflammation. Interestingly, in the case of the latter, eliminating a nociceptive input might reduce the features of central sensitization [137].

On a more clinical note, the prime example of chronic sensitization is fibromyalgia (FM). FM is characterized by chronic widespread pain; normal pressure or heat stimuli might be perceived as nociceptive by the affected individuals. Other commonly observed symptoms are fatigue, sleep, and mood disorders. There is currently a discussion on whether this condition should be regarded as immune-related—studies show that it might indeed have an immune background [138]. IgG from FM patients was shown to cause hypersensitivity to stimuli in mice [138].

Due to the important role of BDNF in the development of central hypersensitization, studies tend to focus on the role of BDNF in the pathophysiology of FM pain. However, as of now, it is difficult to draw conclusions regarding causality in this relation. FM is a heterogenous disease and other common disease symptoms, mentioned above, might also alter the BDNF level, which makes assessments even more difficult.

FM patients were observed to have increased levels of this neurotrophin [139]. However, Bidari et al. have not observed differences in serum BDNF between FM patients and patients with other conditions causing chronic pain [140]. This shows that an increase in serum BDNF is not specific to FM. As expected, therapy with duloxetine instituted in the FM group improved pain as well as decreased BDNF levels [140]. According to another study, high BDNF levels were correlated with a lower pressure pain threshold [141].

Taken together, these studies indicate an important role of BDNF in the development and severity of chronic pain.

Interestingly, physical exercise upregulates BDNF production while decreasing the pain perception [142,143]. As Lee et al. observed, yoga decreased pain severity while increasing serum BDNF [144]. In UC patients, yoga practice decreased the severity of arthralgia [145]. Similarly, in chronic pelvic pain patients, yoga brought about an improvement in pain scores [146]. These results suggest that a greater emphasis needs to be placed on physical activity in patients with immune-related diseases.

Diseases characterized by chronic sleep insufficiency, such as insomnia, tend to feature low levels of BDNF [112]. Counterintuitively, individuals affected with these conditions report more severe pain and a longer pain duration [113]. This example illustrates that pain perception is a complex phenomenon that needs to be perceived as a result of a complex and not yet fully understood set of somatic and psychological processes, rather than a simple occurrence.

In summary, BDNF appears to have an important role in pain experienced by individuals afflicted with immune-related diseases, thus affecting their life quality and increasing the disease burden. Moreover, it might contribute to the greater prevalence of psychiatric disorders, such as depression, anxiety, or sleep disorders, in this population [114,147]. The search for new mechanisms behind pain perception and their use as a therapeutic target is essential, as currently available treatment options, even though effective, still do not fully meet the expectations in many cases [148].

## 6. Influence of Treatment on BDNF

Medications can affect BDNF levels through multiple mechanisms, one of them being the attenuation of inflammation by targeting specific pro-inflammatory cytokines.

Biological anti-TNF therapy with adalimumab (ADA) or infliximab (IFX) has found application in many immune-mediated diseases, such as IBD, RA, ankylosing spondylitis, or psoriatic arthritis. It aims to alleviate inflammation through a reduction in TNF levels. Targeting TNF could also potentially affect BDNF levels, as studies suggest a complex relationship between them.

One study showed that patients with RA had reduced plasma levels of BDNF 14 weeks after treatment with infliximab [92]. This subject has been poorly studied in IBD. As lower levels of BDNF are caused by stress, obesity, and a lack of physical activity, factors considered to affect the etiopathogenesis and course of IBD, results of such studies could contribute to the state of knowledge on this disease [149,150,151]. In one study on IBD patients, 14 weeks of anti-TNF treatment did not affect BDNF levels [152]. However, in this group, BDNF did not correlate with disease severity as measured with the Harvey–Bradshaw Index, which could explain such results. Additionally, disease severity was not objectively assessed during endoscopy or histopathological examination. Thus, the study does not rule out that anti-TNF therapy affects BDNF levels in IBD.

Some studies show the opposite effect of TNF on BDNF [153,154]. Eternacept, another TNF antagonist, was proven to upregulate BDNF production in streptozotocin-induced diabetic mice [155]. In a mouse model of Alzheimer’s disease, adalimumab, apart from exerting neuroprotective and anti-neuroinflammatory effects, was shown to minimize the decline in BDNF [156]. In naïve rats, 8 weeks of IFX administration increased hippocampal and amygdaloid BDNF expression, improving anxiety-like behavior and cognitive functioning [157].

Other biological treatment options targeting different cytokines might also affect the BDNF level [153]. IL-6, a pro-inflammatory interleukin, was shown to increase BDNF secretion in monocytes [153]. Hippocampal and amygdaloid BDNF of mice administered tocilizumab was increased after 8 weeks, with a similar behavioral effect to IFX [157]. IFN (interferon) β treatment, frequently applied in MS, does not seem to change BDNF levels [158]. Natalizumab, a humanized monoclonal antibody against the cell adhesion molecule α4-integrin, used in the treatment of CD and MS, was shown to increase BDNF levels in the latter group, but there are no available studies on this subject in CD patients [159].

Steroids, often used to alleviate inflammation in immune-mediated diseases, have been shown to inhibit BDNF expression in neurons [153]. In a study on SLE patients, neither corticosteroids nor chloroquine diphosphate were shown to influence plasma BDNF, regardless of SLE status (active/inactive) or the presence of neuropsychiatric manifestations [160]. Other studies yielded similar results: corticosteroids, anxiolytics, and antipsychotics did not affect the BDNF level [160]. It is important to remember that dysregulation of the HPA axis, a possible side effect of steroid therapy, might disrupt BDNF expression. A negative correlation between cortisol, the end product of the HPA axis, and BDNF was observed in the prefrontal cortex and cerebrospinal fluid of individuals with schizophrenia [161]. A similar association was present in the sera of drug-naive males with major depression after 3 months of yoga therapy [162].

The influence of other immunosuppressive medications used in the therapy of immune-related diseases is relatively underinvestigated. Cyclosporine A in an animal model was shown to decrease the level of BDNF in the hippocampus and midbrain [163]. Administration of cyclophosphamide had similar effects in the brain [164]. Methotrexate also significantly lowered the BDNF expression in the cortex [165]. Impaired BDNF expression caused by the discussed substances might contribute to their neurotoxic side effects.

Patients with immune-related diseases, among others, due to disease burden and chronic pain, might be more prone to psychiatric disorders, such as depression, which, apart from psychotherapy, often requires pharmacological treatment. BDNF is suspected to be involved in the therapeutic effect of antidepressants [166]. In heterozygous BDNF knock-out mice, a ca. 50% reduction in BDNF levels did not affect depressive behavior. However, it prevented a tricyclic antidepressant (TCA), imipramine, from exerting its effects [166]. Mice with decreased BDNF expression in the forebrain were resistant to the antidepressant action of desipramine, another TCA [166]. In the case of other drugs, according to a meta-analysis that evaluated the effects of sertraline, venlafaxine, paroxetine, and escitalopram, sertraline was the only substance reliably increasing BDNF [167]. There are few studies regarding the effects of fluoxetine, duloxetine, mirtazapine, amitriptyline, and milnacipran, where a BDNF increase was seen; however, researching the mentioned substances would elucidate further this subject [167]. It also appears that BDNF does not predict the outcome of therapy; thus, its use as a biomarker in patients with major depressive disorder remains limited [168].

TNF antagonists have also been used and proven effective in the treatment of depression. Most of the available drugs do not cross the blood–brain barrier. Thus, they might affect the brain only indirectly by attenuating inflammation outside of the CNS, which, according to the inflammatory hypothesis, should help to reduce symptoms of depression. The majority of the available studies on the subject were conducted on individuals afflicted by inflammatory diseases [169]. Here, the contribution of BDNF to the treatment effectiveness is difficult to assess, as this therapy alone alleviates burdensome symptoms of the underlying disease contributing to depression, such as pain. Studies mentioned previously in this paragraph are also ambiguous regarding how anti-TNF drugs affect the BDNF level. Moreover, evidence shows that anti-TNF treatment does not affect mood [127]. Thus, this subject remains highly complex and requires more research to draw final conclusions.

Treatment with BDNF itself is an interesting subject. Several approaches have been developed over the years; however, an effective BDNF drug that would be able to permeate the blood–brain barrier is yet to be invented. Such therapy would be highly sought after in neurological disorders, such as amyotrophic lateral sclerosis or dementia [170]. BDNF might improve remyelination in MS, as well as hinder the cognitive decline seen in this disease. In IBD, BDNF could be able to regulate the function of the enteric glia as well as inhibit apoptosis—SSRI drugs that increase BDNF are already known to alleviate IBD manifestations. Such medication could also improve the quality of life of patients with immune-related disorders by alleviating symptoms of depression or the neurotoxic side effects of certain drugs.

Therapy of patients with immune-mediated diseases might require a complex and individualized approach. The influence of different medications on BDNF is a complex subject to research, as, in many cases, patients need to be administered multiple substances. Thus, the end effect is complicated to assess. More studies on the influence of drugs on BDNF would be desirable, as this neurotrophin is suspected to be implicated in the pathophysiology of conditions comorbid to immune-mediated diseases. Furthermore, based on the available literature, it appears that antidepressant treatment in patients with immune-mediated diseases has a beneficial effect on an individual’s overall clinical state, in part due to its effects on BDNF.

## 7. Conclusions

BDNF has a complex relation to the pathophysiology and symptomatology of immune-related diseases, regulating inflammation, circadian rhythms, psychiatric disorders, and the gut–brain axis. Its role as a neurotrophin with a multifaceted influence on the organism should be further researched in immune-related conditions. More comprehensive knowledge on the subject of BDNF’s contribution to the pathophysiology of the aforementioned diseases will prove useful in the search for novel targets for treatment. Another interesting and active area of research is targeting BDNF in the treatment of pain, particularly chronic pain, hyperalgesia, and allodynia. Advancements in this field have the potential to significantly improve patients’ quality of life and overall well-being.
